# Supplementary material for: Polygyny and intimate partner violence among married women: Sub-national estimates from a cross-sectional study in the Democratic Republic of the Congo
Source: PLOS Glob Public Health. 2025 Jan 3;5(1):e0001645. doi: 10.1371/journal.pgph.0001645 (PMC11698411; doi:10.1371/journal.pgph.0001645)
Supplement: S1 Table — (DOCX) [file pgph.0001645.s002.docx]

Table S1:

Estimated Odds Ratios of the number cowives on intimate partner violence in the Democratic Republic of the Congo by province

|  | Model 1 | | Model 2 | |
| --- | --- | --- | --- | --- |
|  | COR | 95% CI | AOR | 95%CI |
| Province |  |  |  |  |
| Kinshasa | 4.482** | (1.186 - 16.942) | 2.755 | (0.672 - 11.287) |
| Bandundu | 1.471* | (0.977 - 2.216) | 1.683** | (1.013 - 2.794) |
| Kongo Central | 2.264** | (1.108 - 4.628) | 2.217 | (0.559 - 8.795) |
| Equateur | 1.226 | (0.780 - 1.928) | 1.354 | (0.757 - 2.423) |
| Kasai Occidental | 1.033 | (0.747 - 1.430) | 1.000 | (0.704 - 1.421) |
| Kasai Oriental | 0.982 | (0.581 - 1.660) | 0.948 | (0.558 - 1.612) |
| Katanga | 1.217 | (0.836 - 1.772) | 1.467** | (1.019 - 2.112) |
| Maniema | 1.199 | (0.804 - 1.789) | 1.041 | (0.507 - 2.139) |
| North Kivu | 4.334* | (0.907 - 20.718) | 4.693** | (1.065 - 20.680) |
| Orientale | 1.130 | (0.642 - 1.989) | 1.201 | (0.644 - 2.238) |
| South Kivu | 2.659** | (1.112 - 6.360) | 2.776** | (1.060 - 7.272) |
| **All married women** | 1.365*** | (1.100 - 1.691) | 1.302** | (1.034 - 1.639) |
|  |  |  |  |  |
| Statistical significance: | |  |  |  |
| *** p<0.01, ** p<0.05, * p<0.1 | |  |  |  |
