# Supplementary material for: Polygyny and intimate partner violence among married women: Sub-national estimates from a cross-sectional study in the Democratic Republic of the Congo
Source: PLOS Glob Public Health. 2025 Jan 3;5(1):e0001645. doi: 10.1371/journal.pgph.0001645 (PMC11698411; doi:10.1371/journal.pgph.0001645)
Supplement: Attachment — Submitted filename: Point_by_point_responses .docx [file pgph.0001645.s003.docx]

Point-by-point responses

Manuscript: PGPH-D-23-00057
Title: Polygyny and intimate partner violence among married women: Sub-national estimates from a cross-sectional study in the Democratic Republic of the Congo
PLOS Global Public Health


**Section 1 Academic Editor’s comments and questions—Journal Requirements:**

1. Figure 2 is currently divided into multiple panels in separate files. Please include all panels for each figure within a single file, with each panel in the image labelled alphabetically (A, B, C). References to panels within figures should include the figure number and panel letter: e.g., Fig 1A, Fig 1B, Fig 1C. Alternatively, you may wish to separate panels into individual figures, renaming them accordingly (Fig 1A as Fig 1, Fig 1B as Fig 2, etc.).

Please update figure legends and in-text figure citations accordingly.

**Response: Thanks for this comment and suggestion. In the revised version, Figures 1A—1D are now listed as Figs 2—4. Legends have been revised accordingly to reflect these new changes.**

2. Some material included in your submission may be copyrighted. According to PLOS’s copyright policy, authors who use figures or other material (e.g., graphics, clipart, maps) from another author or copyright holder must demonstrate or obtain permission to publish this material under the Creative Commons Attribution 4.0 International (CC BY 4.0) License used by PLOS journals. Please closely review the details of PLOS’s copyright requirements here: PLOS Licenses and Copyright. If you need to request permissions from a copyright holder, you may use PLOS's Copyright Content Permission form.

Please respond directly to this email or email the journal office and provide any known details concerning your material's license terms and permissions required for reuse, even if you have not yet obtained copyright permissions or are unsure of your material's copyright compatibility.

Potential Copyright Issues:

Figures 1 and 2: Please (a) provide a direct link to the base layer of the map (i.e., the country or region border shape) and ensure this is also included in the figure legend; and (b) provide a link to the terms of use / license information for the base layer image or shapefile. We cannot publish proprietary or copyrighted maps (e.g. Google Maps, Mapquest) and the terms of use for your map base layer must be compatible with our CC-BY 4.0 license.

Note: if you created the map in a software program like R or ArcGIS, please locate and indicate the source of the basemap shapefile onto which data has been plotted.

If your map was obtained from a copyrighted source please amend the figure so that the base map used is from an openly available source. Alternatively, please provide explicit written permission from the copyright holder granting you the right to publish the material under our CC-BY 4.0 license.

Please note that the following CC BY licenses are compatible with PLOS license: CC BY 4.0, CC BY 2.0 and CC BY 3.0, meanwhile such licenses as CC BY-ND 3.0 and others are not compatible due to additional restrictions.

If you are unsure whether you can use a map or not, please do reach out and we will be able to help you. The following websites are good examples of where you can source open access or public domain maps: 
* U.S. Geological Survey (USGS) - All maps are in the public domain. ([http://www.usgs.gov](http://www.usgs.gov/)) 
* PlaniGlobe - All maps are published under a Creative Commons license so please cite “PlaniGlobe, [http://www.planiglobe.com](http://www.planiglobe.com/), CC BY 2.0” in the image credit after the caption. (<http://www.planiglobe.com/?lang=enl>) 
* Natural Earth - All maps are public domain. (<http://www.naturalearthdata.com/about/terms-of-use/>)

**Response: We appreciate the rigor the journal applies to ensure that everything included in the manuscript has correct citation or link. In this manuscript, all maps were created using shapefiles of Democratic Republic of the Congo (DRC) from the following website** <https://www.idhsdata.org/idhs/gis.shtml> **and 2013-14 Demographic and Health Survey (DHS) raw data, especially Domestic Violence Module to compute descriptive statistics regarding (1) percentage of women in polygamous marriages; (2) percentage of women who experienced physical violence; (3) percentage of women who experienced emotional violence; (4) percentage of women who experienced sexual violence; and (5) percentage of women who intimate partner violence in the last 12 months.**

3. Please send a completed 'Competing Interests' statement, including any COIs declared by your co-authors. If you have no competing interests to declare, please state "The authors have declared that no competing interests exist".

**Response: “The authors have declared that no competing interests exist”**

4. Please amend your detailed Financial Disclosure statement. This is published with the article. It must therefore be completed in full sentences and contain the exact wording you wish to be published.

If you did not receive any funding for this study, please simply state: “The authors received no specific funding for this work.”

**Response: “The authors received no specific funding for this work”**

5. We have noticed that you have uploaded Supporting Information files, but you have not included a list of legends. Please add a full list of legends for your Supporting Information files after the references list.

**Response: A legend of Supporting Information (SI) has been included after references list.**

Additional Editor Comments (if provided):


[Note: HTML markup is below. Please do not edit.]

Reviewers' comments:

Reviewer's Responses to Questions

**Comments to the Author**

1. Does this manuscript meet PLOS Global Public Health’s [publication criteria](https://journals.plos.org/globalpublichealth/s/criteria-for-publication)? Is the manuscript technically sound, and do the data support the conclusions? The manuscript must describe methodologically and ethically rigorous research with conclusions that are appropriately drawn based on the data presented.

Reviewer #1: Yes

2. Has the statistical analysis been performed appropriately and rigorously?

Reviewer #1: Yes

3. Have the authors made all data underlying the findings in their manuscript fully available (please refer to the Data Availability Statement at the start of the manuscript PDF file)?

The [PLOS Data policy](https://journals.plos.org/globalpublichealth/s/data-availability) requires authors to make all data underlying the findings described in their manuscript fully available without restriction, with rare exception. The data should be provided as part of the manuscript or its supporting information, or deposited to a public repository. For example, in addition to summary statistics, the data points behind means, medians and variance measures should be available. If there are restrictions on publicly sharing data—e.g. participant privacy or use of data from a third party—those must be specified.

Reviewer #1: Yes

4. Is the manuscript presented in an intelligible fashion and written in standard English?

PLOS Global Public Health does not copyedit accepted manuscripts, so the language in submitted articles must be clear, correct, and unambiguous. Any typographical or grammatical errors should be corrected at revision, so please note any specific errors here.

Reviewer #1: Yes

5. Review Comments to the Author

Please use the space provided to explain your answers to the questions above. You may also include additional comments for the author, including concerns about dual publication, research ethics, or publication ethics. (Please upload your review as an attachment if it exceeds 20,000 characters)

**Section 2 Reviewer #1:**

This is a very thoroughly written manuscript on a very sensitive and important topic of intimate partner violence (IPV). The authors have demonstrated extensive knowledge in the subject matter of the paper providing important background literature and a well designed study. The methodology is well prepared and described and the extensive data and sample make the findings very interesting and generalization plausible. The statistical analysis is well conducted and well presented with appropriate tools and tables clearly presenting steps taken to perform the analysis. Again, the authors have taken time and effort to relate and compare the findings to other studies and clearly identified similarities and differences.

There are however, a few issues that I think will help improve the manuscript even better. The first has to do with some level of verbosity of the manuscript. It appears there is verbosity in presenting information such as the study setting, description of the study controls, and some portions of the discussion. Please, address this issue by providing summarized versions of the portions mentioned and other portions.

**Response: We thank the reviewer for this comment. The manuscript has been thoroughly edited and summarized where needed.**

The second issue is that there is a few instances of language or grammatical inconsistency and it will be great to run the manuscript through some form of editing professional or through Grammerly software.

**Response: The manuscript has been thoroughly edited to address this concern**

The third issue is about a statement you made on lines 450-451 in the conclusion about the lawfulness of men marrying more than one wife in DRC. I think this statement may raise concerns of bias, it may seem that polygyny is synonymous to IPV, which is not the case here. It is important to avoid such statements as it can edgy for some reasons, as IPV happens in all kinds of relationships.

**Response: The sentence has been rephrased to address this issue.**

Other than the few issues I have raised, I think this manuscript is very important and well prepared.

6. PLOS authors have the option to publish the peer review history of their article ([what does this mean?](https://journals.plos.org/globalpublichealth/s/editorial-and-peer-review-process#loc-peer-review-history)). If published, this will include your full peer review and any attached files.


**Do you want your identity to be public for this peer review?** If you choose “no”, your identity will remain anonymous but your review may still be made public.


For information about this choice, including consent withdrawal, please see our [Privacy Policy](https://www.plos.org/privacy-policy).

Reviewer #1: No

**Section 3 Reviewer #2:**

Reviewer 2 (Comments sent to Editor over email)

Language
• Language must be factual and non-evaluative. For example, the following types statements are not typical in academic articles:

1. To vehemently question the homogeneity of IPV within countries…
in the Democratic Republic of the
2. …Congo, ironically named the ‘capital of rape’ in the world,

This is not an exhaustive list, but examples of the type of language to edit.

• Language is too colloquyial, like a conversation or an informal write-up. These types of sentences are not appropriate in methods sections “Another issue discussed in multivariable logistic regression is the goodness-of-fit of the estimated models: To what extent do models significantly fit the data?”

• Similarly, “It is worthy mentioning that…”

**Response: We thank the reviewer for raising these issues. The manuscript has been edited and changes are reflected in the revised version.**

Introduction
• The link between IPV and SDGs needs to be drawn out a little more before making this claim, “The high levels of IPV prevalence could be a strong impediment to achieving SDGs by 2030.”

**Response: The first section of the introduction provides a background regarding SDGs, especially those targeting intimate partner violence (IPV).**

• The rationale for why this study explores regional variation in DRC is not totally clear. Similarly, authors hypothesize that polygyny will be associated with IPV but also say this should be different across regions – why? Further, the rationale for a positive linkage between IPV and polygyny, which is mixed in other studies, is not well explained for their prediction in this cultural context.

**Response: We thank the reviewer for the comment. The main argument regarding the relationship between polygyny (or previously lived in polygamous marriages) of married/cohabiting women and intimate partner violence (IPV) is as follows and it is clear in the manuscript:**

- **Most IPV-related adopted country and/or regional approaches and reported finding at national or regional levels. While those studies further our understanding regarding polygyny-IPV relationship, they ignore heterogeneity with countries. In fact, polygyny is not equally distributed within sub-national areas.**
- **By adopting a provincial approach in this study, we unpack the association between polygyny and IPV in the Democratic Republic of the Congo (DRC) on one hand, and on the other hand findings will help policymakers and stakeholders in this area to prioritize since stronger associations between polygyny-IPV means urgent needs to intervene.**
- **We assume that the associations between polygyny and IPV vary because of the acceptance levels of polygyny across provinces and also, because the prevalence of IPV does vary across provinces.**

Method
• The early information presented in the method actually belongs in the introduction on the cultural context for DRC, whereas factual info (e.g., literacy rates of each region sampled) should be in methods. This information still needs to be streamlined, such that only relevant parts that align with the argument presented in the introduction should go in.

**Response: We thank the reviewer for pointing this out. While we agree with the reviewer, it is sometimes important to move in the “Methods” section some useful information which couldn’t be summarized in the introduction to avoid a lengthy introduction.**

• All major claims require robust citations. For example, this sentence is making a claim without citations, so the language needs to be tempered or substantiated with evidence: “However, a latent form of patriarchy still governs these provinces because men have more power than women; these latter have the role of procreation solely to ensure that the families have enough men and women.”

**Response: We thank the reviewer for this comment. The manuscript has now been revised to address this concern.**

• The conceptualization of monogamy is not clear. Just because women denoted no other wives in the family, the unit was considered monogamous? This is not clear whether the expectation and general norm is for the husband to take more wives; please clarify this. It conflates monogamous marriage (by choice) with early marriage (whereby husbands are still able to/can choose to take other wives in the future).

**Response: We thank the reviewer for this comment. The reviewer raises an important point about the concept “polygamy/monogamy”. However, this study uses secondary data analysis and original data was not necessary collected to capture the concept. Therefore, this could be considered a limitation of the study. Responses to the question about the “number of co-wives” are self-reported and can be biased due to lack of information regarding the husbands.**

• Information about other studies and any kins of literature review should not be in the method (e.g., about methodological choices in other studies, rationale for controls etc).

**Response: Although this could be understandable, it is of usual practice in scientific papers to justify in the “Methods section” the choice of the variables included in the analyses.**

• Controls is not labeled right; do you mean covariates? Each exposure here should be a subsection of this section and explained properly.

**Response: We thank the reviewer for raising this issue. “Controls” in this context includes all variables of secondary interest. In this paper, the main interest is the relationship between polygyny and IPV, by province. All other variables included in the analyses are “controls”.**

• Analyses are missing many key details. What were the “spatial variations” used? This is confusing and unclear. Were all 26 districts analysed individually or at regional levels? Much more detail is required about analyses and how it was demarcated, how many N were in each region etc etc.

**Response: In the Methods section, it was stated that the 26 provinces were included in the survey design. However, this paper used the old administrative subdivision which consisted of 11 provinces for two reasons. First, DHS does not allow the calculation of reliable estimates for all outcomes, and that is the case for IPV. Second, and relatedly to the first, not all women were included in the Domestic Violence Module. This reduction of the sample size could lead to unreliable estimates in the 26 provinces given the small sample size (see Table 2).**

• It is not enough to report, “assumptions about logistic regression were carefully checked.” The assumptions are usually explained clearly in a paper.

**Response: This sentence was removed to avoid any misunderstanding for readers.**

Results
• Why are unadjusted odds ratios being presented rather than adjusted ratios?

**Response: We thank the reviewer for this comment. Findings reported consist of binary regression (Unadjusted Odd Ratio—UOR) and multivariate logistic regression (Adjusted Odd Ratio—AOR). See Table 4 (section: Results) and Table S1 (Supporting Information).**

Discussion
• What are the “community-specific effects of polygyny on IPV”? It is unclear what it meant by this and why community-level differences are important to look at. Similarly, variables that could vary by community were not analyzed in this paper, so it is unclear what the theory is that connects the choice of community-level unit to the research topic.

**Response: We thank the reviewer for pointing this out. To address their comment, the paper is interested in the associations between polygyny and IPV at provincial level. This has been revised in the manuscript to use provincial instead of community-specific. However, we use “community-level variables” in the manuscript because, and according to the current literature, IPV is influenced by individual, household, and community-level variables. In this paper, urban residence is an instance of community-level variables. Finally, the approach taken in the paper is highlighted in the last paragraph of the introduction: “To question the homogeneity of IPV within the country, this study adopted a sub-national perspective, taking the province as the unit of interest to unpack the potential inequalities within the country”**

• Examples of interventions at regional levels, that are not being done, should also be in this section.

**Response: Findings from this study are relevant for policymakers and stakeholders in this area when looking into them in terms of (a) exposure and (b) outcome. Assume that the intensity of exposure (POLYGYNY) affects the outcome (IPV), it is clear that all provinces could not be treated equally. Some provinces have higher prevalence of polygyny compared with others. Likewise, some provinces have higher prevalence of IPV compared with others. This information is crucial to devise more effective programmes and interventions.**

• This study used representative sample with survey data collected as part of a national study. However, it is not clear if the information of regional rates of IPV is new or not; presumably the survey authors have already published on IPV prevalence in these provinces? If so, is the novel contribution that this study is analyzing community/regional prevalence by marriage type? The fact of regional variance in itself is not a new finding. We would expect this at all regions in a subnational survey.

**Response: We thank the reviewer for this comment. The added value of this study can be explained in two ways: First, DHS reports are descriptive while this paper reports on logistic models. Second, provincial variations is expected of course; however, this paper fleshed out the foundations of these differences and demonstrated where there are more needs to mitigate the negative effects of polygyny on IPV, while controlling by other relevant variables (controls) found in the literature.**

• Overall, these questions require much more clarification and the unique information contributed by this study to public health is needed.

**Response: We thank the reviewer for pointing this out. Previous studies have clearly demonstrated that IPV is of global concern in both developed and developing concern. Additionally, the role of polygyny has been documented. The specific contribution of the paper is that, by unmasking sub-national differences of the associations between polygyny and IPV, it is possible to tackle this issue where there are more needs.**
