# Supplementary material for: Polygyny and intimate partner violence among married women: Sub-national estimates from a cross-sectional study in the Democratic Republic of the Congo
Source: PLOS Glob Public Health. 2025 Jan 3;5(1):e0001645. doi: 10.1371/journal.pgph.0001645 (PMC11698411; doi:10.1371/journal.pgph.0001645)
Supplement: Attachment — Submitted filename: Comments from review of manuscript PGPH-D-23-00057R1.docx [file pgph.0001645.s004.docx]

Comments from review of manuscript PGPH-D-23-00057R1

- Line 26: Unless you state the year, use indicate, not indicated
- Line 27: Recently? State which regions have been having highest IPV and when SSA started recording the highest IPV (and reasons, if known)
- Lines 28-29 – why is knowing the association between IPV and polygyny important?
- Line 34 and throughout the paper, use of study a bit of a misnomer – that was analysis of DHS data
- Lines 35, 42, 43: background does not bring out the need for this analysis/association
- Lines 47-51, how does knowing the difference in just these 2 parameters contribute or add value?
- Lines 56-59: As presented, the only 2 policy actions would be targeting polygynous families with IPV interventions, or intervene to dismantle polygyny - the latter may not be an option; the former would be tricky and amount to labelling
- Lines 84-85 – a brief explanation of the absence of domestic violence would be helpful
- Line 89 – Peru province is in which country
- Line 91 – IPV prevalence is 30% in which country/region?
- Lines 99-101: while I agree with this, restricting to polygyny vs monogamy may not add as much value as assessing concentrations of IPV and explanatory factors that are amenable to interventions – see comments on lines 56-59
- Lines 130-131: Only parameter tested is polygyny - see earlier comment on opportunity for intervention in polygyny.
- Line 149: …men have more power? Which type? Physical? Economic?
- Line 150: This statement from the authors (not cited) is offensive: [women] have the role of procreation solely to ensure that the families have enough men and women
- Line 155 – be consistent in the use of polygyny vs. polygamy – the author seems to use the terms interchangeably in some places, including table 3
- Lines 158-159: add citation for these documents
- Line 171: citations are numbered – be consistent
- Line 173: what does ‘conceptions of sexuality’ mean?
- Line 178 – data source is 10 years old. Wasn’t there a more recent DHS?
- Line 198 – remove ‘the’ (the marriage)
- Lines 199, 209 – final sample is only women and eligibility includes men; questions on Table 1 imply only women were included in the analysis, so remove definition of eligibility for men
- Lines 228-239: Most of this should be in background, not methods
- Line 249 – final…..move to methods or results if they refer to this paper
- Lines 254-255 – for No – are all the 5 lumped together, or at least one, the way Yes is treated?
- Lines 290-291: Who made the decision to not seek guidance from ethics committee? The authors should have requested for a waiver from an in-country IRB on account of secondary data. By the way (to the editor), what's the journal's policy?
- Line 320: 43% of married women experience violence while prevalence of polygyny is less than half (19%) and now all have experience IPV – this implies other reasons/drivers are equally important and account for more than polygyny so should have strengthened this paper
- Line 338 – married polygynous or monogamous?
- Line 359 – spell out which co-variates were adjusted for?
- Line 369-370 – delete ‘married’ on both lines – redundant
- Line 394 – women’s and child’s – both either plural or singular
- Lines 394-395: How does knowing provincial variations add value
- Line 399: delete the sentence: Second, DRC is one of Central Africa’s countries. – does not add value
- Line 428: word missing between ‘representative’ and ‘to’
- Lines 429-230: This sentence is not clear
- Lines 432-433: what conflates monogamy with early marriage? This means there should have been adjustment for age - it's not clear what was included in the adjustment presented in Table 4
- Line 433-435: Second…… it's not clear what is being communicated here
- Line 435-436 – Third…… was this in the analysis and results? I can't find reference to husband’s characteristics
- Lines 437-438 and 428-439 - combine the 2 sentences in each
- Lines 449-452: it's not clear how knowing that IPV and polygyny can inform policy? What interventions are you having in mind? See earlier comment on Lines 56-59
