# Supplementary material for: Polygyny and intimate partner violence among married women: Sub-national estimates from a cross-sectional study in the Democratic Republic of the Congo
Source: PLOS Glob Public Health. 2025 Jan 3;5(1):e0001645. doi: 10.1371/journal.pgph.0001645 (PMC11698411; doi:10.1371/journal.pgph.0001645)
Supplement: Attachment — Submitted filename: Point_by_point_responses.docx [file pgph.0001645.s005.docx]

**Manuscript PGPH-D-23-00057R1**

**Point-by-point responses to reviewers’ comments**

- Line 26: Unless you state the year, use indicate, not indicated

**Response: This was implemented, using “indicate” instead.**

- Line 27: Recently? State which regions have been having highest IPV and when SSA started recording the highest IPV (and reasons, if known)

**Response: “Recently” was removed in the revised version, and the year was added as suggested.**

- Lines 28-29 – why is knowing the association between IPV and polygyny important?

**Response: This association is important to dig because IPV has several adverse (health, physical, psychological, etc.) outcomes on women**

- Line 34 and throughout the paper, use of study a bit of a misnomer – that was analysis of DHS data

**Response: This is very common in scientific papers. Doing secondary data analysis does not mean it is “NOT” a study. The term “study” is justified because it has a “thesis” and “hypotheses”.**

- Lines 35, 42, 43: background does not bring out the need for this analysis/association

**Response: The following sentence has been added in the background “Additionally, previous research showed that polygyny is positively associated with IPV” to stress out the importance to study the association between polygyny and IPV, and specifically the need to understand this association at lower geographical level.**

- Lines 47-51, how does knowing the difference in just these 2 parameters contribute or add value?

**Response: Thanks for the comments; however, we couldn’t elaborate more on this summary. The authors reported on the “main findings” and provided more details through the manuscript. It is clear from the findings that (i) there are geographical variations of IPV in the country; and (ii) the effects of polygyny varied across provinces. This has policy and programmatic implications for stakeholders and policymakers working on this area.**

- Lines 56-59: As presented, the only 2 policy actions would be targeting polygynous families with IPV interventions, or intervene to dismantle polygyny - the latter may not be an option; the former would be tricky and amount to labelling

**Response: Thanks for comments and suggestions. To be more practical, findings/research might recommend but researchers won’t be on the ground to eradicate polygyny nor implement IPV interventions. Modestly, researchers suggest/recommend based on research’s findings. Again, findings showed geographical variations of (i) IPV rates and (ii) the associations between polygyny and IPV which mimic the prevalence of polygyny in the country. Therefore, resources might be allocated in more prevalent provinces in terms of polygyny and IPV rates through Information—Education—Communicaton (IEC) campaigns regarding the adverse outcomes of IPV and the exacerbating power of polygyny.**

- Lines 84-85 – a brief explanation of the absence of domestic violence would be helpful

**Response: Thanks for the comments. An explanation has been added at the end of the sentence (in red) which now reads “For instance, research suggest that domestic violence was virtually absent in small-scale societies like the Wape of Papua New Guinea, partly because the Government promotes egalitarian rules between women and men.”**

- Line 89 – Peru province is in which country

**Response: This is located in Peru. Adding “Peru” in the same sentence might be redundant.**

- Line 91 – IPV prevalence is 30% in which country/region?

**Response: The sentence has been clarified to reflect that estimates are worldwide and reads now “For example, while the overall IPV prevalence was estimated at 30% worldwide.”**

- Lines 99-101: while I agree with this, restricting to polygyny vs monogamy may not add as much value as assessing concentrations of IPV and explanatory factors that are amenable to interventions – see comments on lines 56-59

**Response: Thanks for your comments and we appreciate your way of thinking. Each paper is in fact a “thesis”. This paper is interested in the relationship between polygyny and IPV. This does not mean that other (individual, community, and structural) factors aren’t important at all. In Table 4, bivariate (or crude effect) and multivariate (net effect) findings are reported. It means that the associations between polygyny and IPV were controlled for other factors. However, to keep the readers concentrated by the main thesis of the paper “associations between polygyny and IPV” at national and provincial level, only associations between polygyny and IPV are consistently reported. This is also common in scientific papers.**

- Lines 130-131: Only parameter tested is polygyny - see earlier comment on opportunity for intervention in polygyny.

**Response: Thanks for the comments and kindly refer to previous response for similar comment.**

- Line 149: …men have more power? Which type? Physical? Economic?

**Response: Thanks again for the comments. The sentence was clarified as follows: “However, a latent form of patriarchy still governs these provinces because men have more political power, moral authority and control of property than women.”**

- Line 150: This statement from the authors (not cited) is offensive: [women] have the role of procreation solely to ensure that the families have enough men and women

**Response: Thanks for the comments. To align with reviewers’ comments, the sentence was removed in the revised version.**

- Line 155 – be consistent in the use of polygyny vs. polygamy – the author seems to use the terms interchangeably in some places, including table 3

**Response: Thanks again for the comments. To incorporate this comment, we used consistently polygyny or polygynous unions/marriages in the revised version.**

- Lines 158-159: add citation for these documents

**Response: Thanks for this comment. References to documents were added in the revised version.**

- Line 171: citations are numbered – be consistent

**Response: Thank you. This comment was incorporated.**

- Line 173: what does ‘conceptions of sexuality’ mean?

**Response: Thanks for the comment. To avoid any confusion, this was removed in the revised version.**

- Line 178 – data source is 10 years old. Wasn’t there a more recent DHS?

**Response: The 2013—2014 DHS is the most recent available data in the DR Congo. A recent DHS was conducted in 2022; however, data are not available yet.**

- Line 198 – remove ‘the’ (the marriage)

**Response: As recommended, “the” has been deleted.**

- Lines 199, 209 – final sample is only women and eligibility includes men; questions on Table 1 imply only women were included in the analysis, so remove definition of eligibility for men

**Response: As recommended, men were excluded. However, the original idea was that data were collected on men (15—59 years) and women (15—49 years) in the DHS.**

- Lines 228-239: Most of this should be in background, not methods

**Response: We agree with the comments; however, it is not unusual to proceed this way in published papers in PLOS ONE. The idea is that the background section focuses on key ideas and hypotheses to be tested. In lines 228—239, the idea is “to fully justify the inclusion” of control variables.**

- Line 249 – final…..move to methods or results if they refer to this paper

**Response: This was deleted and replaced by “Previous studies also found that attitudes towards violence were associated with IPV”.**

- Lines 254-255 – for No – are all the 5 lumped together, or at least one, the way Yes is treated?

**Response: out of 5 reasons to justify violence, a woman is coded 0 is she responded NO on all five reasons. Those women with a score 1 or higher on five items were coded 1.**

- Lines 290-291: Who made the decision to not seek guidance from ethics committee? The authors should have requested for a waiver from an in-country IRB on account of secondary data. By the way (to the editor), what's the journal's policy?

**Response: This is a secondary data analysis. Ethics approval is required for primary data collection. In this case, data were anonymized by the DHS program and therefore, Ethics approval was not required. Papers using DHS datasets contain such statement.**

- Line 320: 43% of married women experience violence while prevalence of polygyny is less than half (19%) and now all have experience IPV – this implies other reasons/drivers are equally important and account for more than polygyny so should have strengthened this paper

**Response: We totally agree with this comment; however, the paper has a focus “Polygyny and IPV at national and sub-national levels”. The paper included “controls” and devoted special attention to justify the inclusion of these controls. This also means that the “paper doesn’t ignore the importance of these factors”. Readers will find that, in multivariate findings, estimates control for other putative covariates, even though the paper didn’t report specifically on those “controls”.**

- Line 338 – married polygynous or monogamous?

**Response: This was corrected according to reflect married women in polygynous unions.**

- Line 359 – spell out which co-variates were adjusted for?

**Response: The footnotes (Table 4) clearly indicate the list of variables included in the estimations.**

- Line 369-370 – delete ‘married’ on both lines – redundant

**Response: This has been deleted as recommended.**

- Line 394 – women’s and child’s – both either plural or singular

**Response: For consistency, we kept “singular” for both and reads now “woman’s and child’s health”**

- Lines 394-395: How does knowing provincial variations add value

**Response: Findings indicate that provinces are unevenly affected by polygyny and IPV. Therefore, IPV interventions in the DRC should devote more efforts to most affected provinces. This can only be achieved with this kind of analyses.**

- Line 399: delete the sentence: Second, DRC is one of Central Africa’s countries. – does not add value

**Response: This has been deleted as recommended.**

- Line 428: word missing between ‘representative’ and ‘to’

**Response: The sentence reads now “This study used a nationally representative dataset to examine the association between polygyny and IPV.”**

- Lines 429-230: This sentence is not clear

**Response: Original sentence “This is a strength because the original sampling design aimed at producing valid estimates at subnational level”**

**Revised sentence: “Indeed, the original sampling design aimed at producing valid estimates at subnational level”**

- Lines 432-433: what conflates monogamy with early marriage? This means there should have been adjustment for age - it's not clear what was included in the adjustment presented in Table 4

**Response: In multivariate findings, estimates control for other putative covariates, even though the paper didn’t report specifically on those “controls”.**

- Line 433-435: Second…… it's not clear what is being communicated here

**Response: The sentence was deleted.**

- Line 435-436 – Third…… was this in the analysis and results? I can't find reference to husband’s characteristics

**Response: Since they weren’t included in the analyses, it is a limitation.**

- Lines 437-438 and 428-439 - combine the 2 sentences in each

**Response: This was done as recommended.**

- Lines 449-452: it's not clear how knowing that IPV and polygyny can inform policy? What interventions are you having in mind? See earlier comment on Lines 56-59

**Response: Findings showed that the prevalence of IPV in the DRC mimicked the distribution of polygyny. As a consequence, policymakers and stakeholders couldn’t ignore polygyny if they want to get long-term positive effects when designing IPV interventions.**
