# Supplementary material for: Polygyny and intimate partner violence among married women: Sub-national estimates from a cross-sectional study in the Democratic Republic of the Congo
Source: PLOS Glob Public Health. 2025 Jan 3;5(1):e0001645. doi: 10.1371/journal.pgph.0001645 (PMC11698411; doi:10.1371/journal.pgph.0001645)
Supplement: Attachment — Submitted filename: Re-review of PGPH-D-23-00057R2.docx [file pgph.0001645.s006.docx]

**Re-review of PGPH-D-23-00057R2 (Polygyny and intimate partner violence among married women: Sub-national estimates from a cross-sectional study in the Democratic Republic of the Congo)**

Grateful to the authors for addressing most of my previous concerns raised in the first review except in one or 2 places (see comments). I have very few, very minor, comments.

**Abstract**:

Line 45: ‘components’ – consider replacing with ‘manifestations’ or some other term

**Introduction**:

Lines 93-94: The difference between 36% and 30% is small so phrases like ‘as high as’ or the ‘high levels of IPV’ not supported by provided data. Perhaps tone down the wording. Also term ‘drastic’ on line 95 – too strong

Line 104 – ‘to’ polygyny

Line 106: ‘To provide with insights on IPV’ not clear what is meant – maybe remove ‘with’?

107-108 – cite the framework immediately after, separate from its description

117 – not necessarily ‘many’; more than one wife is enough to qualify as polygynous

**Methods**:

150-151: Except for the province of Kinshasa, the Capital City of the country, the DRC is

subdivided into matrilineal societies (South Bandundu and Kongo central)….sentence sounds incomplete.

160-176 – more like background material – same comment made at first review

Line 180 – please provide citation for the DHS. Same comment made at initial review

Line 206 – ‘component’ – comment as previous

222-225: reads like background info

**Results**:

Line 321 – respectively does not apply
