# Supplementary material for: Polygyny and intimate partner violence among married women: Sub-national estimates from a cross-sectional study in the Democratic Republic of the Congo
Source: PLOS Glob Public Health. 2025 Jan 3;5(1):e0001645. doi: 10.1371/journal.pgph.0001645 (PMC11698411; doi:10.1371/journal.pgph.0001645)
Supplement: Attachment — Submitted filename: Review comments.docx [file pgph.0001645.s007.docx]

The paper discusses an important topic of intimate partner violence and also assess the regional variation of IPV among polygyny women, but there are few things which can be improved.

**Introduction**

Comment 1: The author needs to explain why they are focusing on women in polygyny relationships in the context of intimate partner violence.

Comment 2: Since the author has mentioned SDG 5.2, they need to elaborate on the aim and target of SDG 5 and explain its relevance to their study.

**Method**

Comment 3: Lines 145-149 need to be restructured to clearly explain how the type of society and geographical location impact intimate partner violence.

Comment 4: Lines 220-226, can be move to literature review section under the introduction heading.

Comment 5: The author should have mentioned the value VIF in order to reveal that there was no multicollinearity in the study.

**Discussion**

Comment 5: The finding needs to be discussed briefly. There should be brief discussion of what are the significant findings, how they align with the previous studies and what could be the possible reason for the observed result.

Comment 6: Author have mentioned about patrilineal and matrilineal societies effect on intimate partner violence, but author do not mention how the prevalence of intimate partner violence among polygyny differs across these two societies.
