# Supplementary material for: Polygyny and intimate partner violence among married women: Sub-national estimates from a cross-sectional study in the Democratic Republic of the Congo
Source: PLOS Glob Public Health. 2025 Jan 3;5(1):e0001645. doi: 10.1371/journal.pgph.0001645 (PMC11698411; doi:10.1371/journal.pgph.0001645)
Supplement: Attachment — Submitted filename: Point-by-point-responses-R3.docx [file pgph.0001645.s008.docx]

**Point-by-point responses**

**PGPH-D-23-00057R2**

**Title: Polygyny and intimate partner violence among married women: Sub-national estimates from a cross-sectional study in the Democratic Republic of the Congo**

PLOS Global Public Health

6. Review Comments to the Author

Please use the space provided to explain your answers to the questions above. You may also include additional comments for the author, including concerns about dual publication, research ethics, or publication ethics. (Please upload your review as an attachment if it exceeds 20,000 characters)

Reviewer #2: I am unable to respond to availability of data (no 4 above) so response is place holder. Maybe 'don't know' option should be included for reviewers.

**Response: Thank you for raising the issue about “data availability”. This study is an “analysis of secondary data (2013—2014 Demographic and Health Survey (DHS), a nationally representative survey) to answer a specific question: “Does polygyny (referred to as marriage between a man and 2 wives or more) correlate with intimate partner violence (IPV)” in the Democratic Republic of Congo, with specific attention on sub-national estimates. In doing so, the present study departs from previous studies (**[**https://www.sciencedirect.com/science/article/pii/S0304387818314810**](https://www.sciencedirect.com/science/article/pii/S0304387818314810)**;** [**https://onlinelibrary.wiley.com/doi/epdf/10.1111/jomf.12570**](https://onlinelibrary.wiley.com/doi/epdf/10.1111/jomf.12570)**). While other putative factors were found to be significantly associated with IPV, this paper focuses on “polygyny” while controlling for relevant factors (education, age, household wealth index, etc.).**

Comments are as below:

Re-review of PGPH-D-23-00057R2 (Polygyny and intimate partner violence among married women: Sub-national estimates from a cross-sectional study in the Democratic Republic of the Congo).

Grateful to the authors for addressing most of my previous concerns raised in the first review except in one or 2 places (see comments).

**Response: Thank you to mentioning this.**

I have very few, very minor, comments.
Abstract:
Line 45: ‘components’ – consider replacing with ‘manifestations’ or some other term

**Response: We have replaced it by the “three types” and sentence reads now:**

**“There were significant variations across provinces in the Democratic Republic of the Congo for polygyny and the three types of IPV, including physical, emotional, and sexual violence”**

Introduction:
Lines 93-94: The difference between 36% and 30% is small so phrases like ‘as high as’ or the ‘high levels of IPV’ not supported by provided data. Perhaps tone down the wording. Also term ‘drastic’ on line 95 – too strong

**Response: We have revised the sentence which reads now “its prevalence in SSA was estimated at 36%”**

Line 104 – ‘to’ polygyny

**Response: Thank you for suggestion**

Line 106: ‘To provide with insights on IPV’ not clear what is meant – maybe remove ‘with’?

**Response: We have rephrased the sentence which reads now “To further our understanding on IPV worldwide, researchers have devoted much time to find the putative correlates of IPV”**

107-108 – cite the framework immediately after, separate from its description

**Response: Thank you for this suggestion and the sentence reads “Drawing on Bronfenbrenner’s ecological framework, some studies posited that correlates of IPV are found at individual/personal, household, and community levels.”**

117 – not necessarily ‘many’; more than one wife is enough to qualify as polygynous

**Response: Thank you for this suggestion which has been incorporated.**

Methods:

150-151: Except for the province of Kinshasa, the Capital City of the country, the DRC is
subdivided into matrilineal societies (South Bandundu and Kongo central) …. sentence sounds incomplete.

**Response: Thanks for the suggestion. We have accordingly revised the sentence.**

160-176 – more like background material – same comment made at first review

**Response: Thank for this comment. However, kindly note that the background (Introduction) focused on theoretical and empirical evidence on the relationships between polygyny and IPV. This information (160—176) offers the readers additional information to better understand the relationship between polygyny and IPV in the context of DRC.**

Line 180 – please provide citation for the DHS. Same comment made at initial review

**Response: We have provided the reference. Thank you**

Line 206 – ‘component’ – comment as previous

**Response: We have replaced component by “three types of IPV”.**

222-225: reads like background info

**Response: Thank for this comment. It is sometimes important to provide “references” so that the readers know that independent variables were drawn from previous studies.**

Results:
Line 321 – respectively does not apply

**Response: Thank you. The word “respectively” was removed.**

Reviewer #3

This manuscript reported Polygyny and IPV among married women in the Democratic Republic of the Congo. This study brings some essential facts, however, there are some points I would like the authors to address to further strengthen the manuscript.

Abstract
1. The authors are suggested to kindly maintain one form of tense in the background.

**Response: Thank you for the comments.**

2. Can the authors clarify, what they want to mean by differentials in the conclusion?

**Response: Thank you. The word “differentials” was replaced by “different”.**

3. Is there any policy introduced by the government to lessen IPV among women in DRC? If yes, then the authors are requested to mention those.

**Response: Thank you for the comment.**

Introduction
4. Line 77, when it has already been stated in SDG, there is no need to put for instance.

**Response: Thank you for the comment. This sentence was removed in the revised version.**

5. Lines 78-80, the authors are requested to put intext citations, since, its stating that previous studies have concluded.

**Response: Thank you for the comment. This sentence has been revised to remove “Previous studies…”**

6. Line 86, can the authors specific what they want to intend to the readers by small scale societies?

**Response: This expression is used to describe “small societies. See for instance:** [**https://www.sciencedirect.com/science/article/abs/pii/S0020729202000383**](https://www.sciencedirect.com/science/article/abs/pii/S0020729202000383)**.**

7. Line 92, authors can do away writing, “for example”, since you are stating a fact, there is no need of writing this as an example.

**Response: The sentence was revised and reads now “Indeed, the overall IPV prevalence was estimated at 30% worldwide while it was estimated at 36% in SSA”.**

8. Line 98, The regions referred here, is it the study region?

**Response: We have clarified as “world’s regions”.**

9. The overall introduction provides some missing links to the readers. For e.g. The 3rd and 4th paragraph of page 5, do not show any interconnection. I rather suggest the authors, to kindly arrange the paragraphs of the introduction in a more story telling manner, which will help the readers to understand better.

**Response: We have clarified to help to improve the flow of the “Introduction”.**

10. Authors are requested to kindly provide with intext citations in Line 118-120.

**Response: References are fully provided before summarizing the findings of these studies (See Lines 120—133).**

11. Line 124, do the authors mean to say, that the study is based on data collected from 16 countries, to show inequality among IPV within the democratic republic of Congo? Please clarify.

**Response: This sentence refers to reference [10]. “This study built on nationally representative data from 16 countries to provide insights into the intricacies between polygyny and IPV at the national level, therefore masking the inequalities within countries”.**

**We have slightly rephrased the sentence.**

12. I think, it will be beneficial for the readers if the authors clearly mention the research/study gap of this study?

**Response: Following previous comment, the study clearly indicates that studies between polygyny and IPV at national level mask variations at sub-national levels. The present study fills this gap in analyzing the relationship between polygyny and IPV at sub-national levels.**

Data
13. I think the authors can think about putting study setting in the data section, as this paragraph does not imply to data and data related information of the study. The study setting is explaining IPV in different regions of DRC.

**Response: It is common that “Methods” section starts with “Study setting” as we did in this paper.**

14. Line 232 and 233 are inferring to same meaning. Can the authors kindly write about the level of education in one sentence to give a complete meaning?

**Response: Thank you for the comment. Additional sentence was removed.**

15. Line 240, Can the authors clarify what they intend to signify by stating that it has been published elsewhere? They are requested to give a complete information.

**Response: Thank you for the comment. Reviewers might have noted that this sentence has references. It is very common in scientific papers to see this kind of sentences.**

16. Can the author mention the value of VIF, while stating no multicollinearity existed? Also, for goodness of fit of the models, can the authors mention the value of R2?

**Response: Thank you for the comment. The sentence has been completed as follows: “Overall, VIF was 1.35 and it ranged from 1.02 to 1.81.” regarding R^2^, since we are dealing with logistic modeling, this statistic is less relevant.**

17. Can the authors provide a table showing the prevalence of all independent and control variables of the study population? For e.g. Prevalence rates of poorest, poorer, richer, and richest among the study population?

**Response: Thanks for the comments and suggestion. As mentioned above, we all know that social issues are total and global. That said, not a single variable (polygynous unions in this study) can explain a social issue. However, the help policymakers and stakeholders, this study has focused on “polygyny” and its association with IPV.**

Discussion
18. I found the discussion of the study as a reiteration of same facts. I would rather suggest, the authors if they incorporate the associations of other variables such as level of education, poverty with IPV along with polygyny, the study will give a better read.

**Response: Thank for the comment. Please refer to the response on comment #17. While recognizing the importance of “other variables” this paper focused on “polygyny” and its association with IPV.**

Reviewer #5: The manuscript titled "Polygyny and Intimate Partner Violence Among Married Women: Sub-National Estimates from a Cross-Sectional Study in the Democratic Republic of the Congo" explores the relationship between polygyny and intimate partner violence (IPV) among married women. The study utilizes data from the Demographic and Health Survey (DHS) 2013-2014. The study aims to provide a detailed understanding of how polygyny correlates with various forms of IPV—physical, emotional, and sexual—across different provinces in the DRC. The manuscript claims that women in polygynous unions are at a higher risk of experiencing IPV compared to those in monogamous marriages. Additionally, it highlights significant spatial variations in the prevalence of both polygyny and IPV, suggesting the need for region-specific interventions.

**Response: Thank you for this encouraging comment.**

Strengths
Relevance and Contribution: The manuscript addresses a pressing public health and social issue in the DRC, contributing valuable insights into the complex dynamics between marital structure and IPV. The focus on sub-national estimates is particularly commendable, as it uncovers important regional disparities that can inform targeted policy responses.

**Response: Thank you for this encouraging comment.**

Methodological Approach: The use of DHS data and multivariate logistic regression to analyze the relationship between polygyny and IPV provides a robust and credible foundation for the study. The data analysis is well-executed, with the clear and coherent presentation of results, supported by appropriately detailed tables and figures.
Clarity and Organization: The manuscript is generally well-organized, with a logical flow that facilitates understanding. The results are presented clearly, making it easy to follow the narrative from the introduction through to the discussion.

**Response: Thank you again for this encouraging comment.**

Weaknesses
Contextual Gaps: The introduction would benefit from a more comprehensive contextualization of the socio-cultural factors that contribute to the prevalence of polygyny in the DRC.

**Response: Thank you for this comment; however, the space for such exercise may be quite difficult. We have provided much information in “Study setting” sub-section to help readers better understand the social landscape in the country.**

Insufficient Exploration of Regional Variations: While the study provides important data on regional variations, the discussion section could be strengthened by delving deeper into the potential causes of these differences. Providing a more nuanced analysis of why certain regions exhibit higher rates of polygyny and IPV would enhance the manuscript's overall impact.

**Response: Thank you for the comment. However, the authors don’t want to fall in “non-explored facts” in the sense that “this is a secondary data analysis” and other relevant factors to better understand provincial disparities might be difficult to prove with secondary data analysis.**

Policy Implications: The discussion of policy implications is somewhat underdeveloped. Given the strong association between polygyny and IPV, the manuscript could benefit from more concrete recommendations for interventions or policies aimed at reducing IPV in regions with high polygyny rates.

**Response: Thank you for the comment. The manuscript is clear “Policymakers and stakeholders should integrate polygyny when devising interventions and programs to fight IPV”.**

Limitations and Specificity: The limitations section could be expanded to include a more detailed discussion of the challenges associated with using cross-sectional data, particularly regarding the potential for underreporting of IPV. Moreover, the manuscript would benefit from a clearer articulation of the specific gaps in the literature that this study aims to fill.

**Response: Thank you for the comment. The “limitations” section already addressed “reporting bias” which might result in social desirability about IPV. We have added a sentence about “causality” between polygyny and IPV.**

Recommended Course of Action
The manuscript is a valuable contribution to the literature on IPV and polygyny, particularly within the context of the DRC. However, I recommend revisions to address the contextual and theoretical gaps, explore regional variations more thoroughly, and strengthen the discussion of policy implications. Expanding the limitations section and clarifying the study's specific contributions to the existing literature would also be beneficial.

Abstract
• Lines 39: Specify the putative factors adjusted for in the analysis to provide clarity.

**Response: Thank you for the comment. In Table 4, Model 2 controls for women’s education, age, urban residence, household’s wealth index, and attitudes towards intimate partner violence (see page Table 4, page 18).**

• Lines 49-50: Simplify the language by specifying the nature of the variations, e.g., "Kasai Occidental had higher rates of IPV."

**Response: Thank you for the comment. However, this precision is important since the paper is interested in sub-national estimates.**

• Lines 56-57, 442-443: Summarize the key findings more explicitly in the conclusion, mentioning which provinces had the highest rates of IPV and the overall impact of polygyny.

**Response: Thank you for the comment. This comment was incorporated in the conclusion (Abstract) and as suggested by the reviewer.**

• Lines 58-59: Mention specific interventions or policy changes that could strengthen the impact of the conclusion.

**Response: The conclusion clearly mentioned that “policymakers and stakeholders should take into account polygyny to devise more effective interventions against IPV”. However, since polygyny is culturally rooted in African societies and DRC as well, it is difficult to suggest “specific” IPV interventions, at least in the short run.**

Introduction
• Line 73: Use "predominantly directed at" instead of "mostly against."

**Response: Thank for the comment. However, this is a citation. See the reference.**

• Line 74: Replace "attention in" with "attention over."

**Response: Thanks for the comment. This was changed.**

• Lines 75-78: Merge these lines into one sentence for clarity, transitioning from general to specific (SDG to SDG 5.2).

**Response: Thank you. This was changed. The sentence “For instance, SDG 5.2 aims to eliminate all forms of violence against women and girls in public and private spheres by 2030” was deleted.**

• Line 90: Use "Data suggests" instead of "available data suggests."

**Response: Thank you. This was incorporated.**

• Line 93: Replace "overall and worldwide" with more comprehensive terms like "the global prevalence of IPV."

**Response: Thank you. This was incorporated.**

• Lines 89-94: Reorganize for a more logical flow—start with the global context, move to regional specifics, then discuss the DRC.

**Response: Thank you. This was incorporated.**

• Line 94: Use "these high levels" instead of "the high levels."

**Response: Thank you. This was incorporated.**

• Line 95: Replace "unless drastic measures" with a more prescriptive phrase like "necessitating urgent action."

**Response: Thank you. This was incorporated. The sentence reads now “These high levels of IPV prevalence could make it difficult for the subregion to achieve the SDGs by 2030, unless measures necessitating urgent action are implemented across SSA and within countries [15].”**

• Line 106: Instead of "the researcher in the context," use "the research context," focusing on how research has explored various drivers of the issue.

**Response: Thank you. This was incorporated.**

• Line 118: Include specific findings from key studies, such as how much more prevalent IPV is in polygynous unions compared to monogamous ones.

**Response: Thank you. This was incorporated.**

• Lines 126-129: Explicitly state the gaps in the literature that this study aims to fill.

**Response: Thank you. This was incorporated.**

• Line 129: Consider stating "To investigate the homogeneity or heterogeneity..."

**Response: Thank you. This was incorporated. Homogeneity was replaced by “heterogeneity”**

• Line 132: Use "expected to provide" instead of "provide."

**Response: Thank you. This was incorporated.**

Methods
• Lines 145-147: To strengthen the theoretical framework, include information on how patriarchal norms differ in matrilineal vs. patrilineal societies and how these structures influence polygyny and IPV prevalence.

**Response: Thank you. This was incorporated. A sentence was added: “Previous research clearly suggests that relationships between societal structure and IPV are very complex. While patrilineal societies may reinforce power imbalances that contribute to IPV, matrilineal societies can promote female empowerment and community support, potentially reducing violence. However, other factors (e.g., socio-economic conditions, legal frameworks, and cultural attitudes toward violence) could influence these dynamics. Understanding these nuances is crucial for developing effective prevention and intervention strategies.” See page 7)**

• Line 157: Use "into the mapping" instead of "on the mapping."

**Response: Thank you. This was incorporated**

• Line 265: Include details on how missing data was handled, particularly regarding IPV reporting, which often suffers from underreporting.

**Response: Thank you. This was incorporated**

Results
• Line 301: Given the focus on polygyny and IPV, including the prevalence of polygyny and the sample size of provinces in Table 2 would be more comprehensive and supplement Figure 1.

**Response: Thank you. This was incorporated in Table 2, and Table caption revised accordingly.**

• Consider separating the description of prevalence rates and spatial variations of IPV into distinct subsections for clarity.

**Response: Thank you for this suggestion. However, given they are short, it is meaningless to add sub-headings.**

• Lines 339-346: Explore the reasons behind these variations to add depth to the analysis.

**Response: Thank you. In this section, we present “results”. Further information was included later in “Discussion” section.**

• Lines 379-384: Provide a more detailed explanation of the implications of the AORs presented in Table 4, particularly for provinces like Kongo Central, where the AOR was marginally significant. Clarify whether these findings suggest a trend or require cautious interpretation due to wide confidence intervals.

**Response: Thank you. We agree that these findings should be interpreted with caution given the small sample size in some provinces, including Kongo central (see Table 2)**

Discussion
• Lines 418-419: Expand the discussion on the policy implications of the findings, particularly how understanding sub-national variations can inform targeted interventions. Discuss specific strategies that could be implemented at the provincial level to address IPV in regions with high polygyny rates.

**Response: Thank you. This was incorporated in “Discussion” section already.**

Conclusion
• Line 442: Use "show" or "reveal" instead of "showed."

**Response: Thank you. This was incorporated in the revised version.**

• Lines 448-452: Strengthen the conclusion by incorporating specific recommendations for actionable steps, acknowledging potential limitations, and emphasizing the need for localized interventions. Suggest that future research explore additional factors, such as male partner characteristics and community-level influences, to deepen our understanding of IPV drivers in different contexts.

**Response: Thank you. This was incorporated in the revised version. In the conclusion: “Policymakers and stakeholders should devise interventions and policies that target these intertwined issues taking into the specificities of each province. Therefore, there is an urgent need for localized interventions to tackle IPV. Future research should also explore additional factors, such as husband/partner’s characteristics and community-level influences, to further our understanding of IPV drivers in different contexts.”**

Other Suggestions
• Improve the clarity and comprehensiveness of table and figure labels.

**Response: Thank you. This was incorporated in the revised version.**

• Provide a clear and operational definition of IPV specific to this study's universe.

**Response: Thank you. This was incorporated in the revised version.**

• Language editing is required.

**Response: Thank you. This was incorporated in the revised version.**

Reviewer #3:

The paper discusses an important topic of intimate partner violence and also assess the regional variation of IPV among polygyny women, but there are few things which can be improved.

**Response: Thank you.**

Introduction

Comment 1: The author needs to explain why they are focusing on women in polygyny relationships in the context of intimate partner violence.

**Response: Thank you. This is explained in the “Introduction” section. Polygyny, a marriage between a man and 2 wives or more has been associated with social and health consequences.**

Comment 2: Since the author has mentioned SDG 5.2, they need to elaborate on the aim and target of SDG 5 and explain its relevance to their study.

**Response: Thank you. This was incorporated in the revised version.**

Method

Comment 3: Lines 145-149 need to be restructured to clearly explain how the type of society and geographical location impact intimate partner violence.

**Response: Thank you. This was incorporated in the revised version.**

Comment 4: Lines 220-226, can be move to literature review section under the introduction heading.

**Response: Thank you. However, the paper has a “focus” which is polygyny. Therefore, it is normal that other information be managed accordingly as we did.**

Comment 5: The author should have mentioned the value VIF in order to reveal that there was no multicollinearity in the study.

**Response: Thank you. This was incorporated in the revised version.**

Discussion

Comment 5: The finding needs to be discussed briefly. There should be brief discussion of what are the significant findings, how they align with the previous studies and what could be the possible reason for the observed result.

**Response: Thank you. This was incorporated in the revised version.**

Comment 6: Author have mentioned about patrilineal and matrilineal societies effect on intimate partner violence, but author do not mention how the prevalence of intimate partner violence among polygyny differs across these two societies.

**Response: Thank you. This was incorporated in the revised version.**

**“Previous research clearly suggests that relationships between societal structure and IPV are very complex. While patrilineal societies may reinforce power imbalances that contribute to IPV, matrilineal societies can promote female empowerment and community support, potentially reducing violence. However, other factors (e.g., socio-economic conditions, legal frameworks, and cultural attitudes toward violence) could influence these dynamics. Understanding these nuances is crucial for developing effective prevention and intervention strategies.” (see p.7).**
