# Supplementary material for: Polygyny and intimate partner violence among married women: Sub-national estimates from a cross-sectional study in the Democratic Republic of the Congo
Source: PLOS Glob Public Health. 2025 Jan 3;5(1):e0001645. doi: 10.1371/journal.pgph.0001645 (PMC11698411; doi:10.1371/journal.pgph.0001645)
Supplement: Attachment — Submitted filename: Point-by-point-responses-R4.docx [file pgph.0001645.s009.docx]

**Point-by-point responses**

**PGPH-D-23-00057R2**

**Title: Polygyny and intimate partner violence among married women: Sub-national estimates from a cross-sectional study in the Democratic Republic of the Congo**

PLOS Global Public Health

Additional Editor Comments (if provided):

1. I would like to request more detail regarding the analysis reported in table 3. Earlier in the manuscript (in the methods section), you mention that "Statistical significance was tested at national and sub-national levels using Pearson’s chi-square tests", but there is no description of the tests performed in the results section.

**Refer (response 1)** I assume that the p values reported in table 3 are from chi-square analyses, but I cannot be sure. Also, I can see how the association between province and each of the four dependent variables can be assessed using chi-square (i.e., the bolded p values at the bottom of the table), but it is not clear how the p values for each province were derived. For example, you report a non-significant association between marital status and physical violence in Kinshasa: Is this a test comparing the score of 13.8 and the score of 25.7? What test was used? Or was this a test using the underlying data rather than the means?

**Refer (response 2)** Also, I believe that the numbers reported in the table are percentages, but this information is not stated. In short, please provide a lot more information about how the data were analysed.

Please review our guidelines for statistical reporting and ensure your manuscript complies with these (<https://journals.plos.org/globalpublichealth/s/submission-guidelines#loc-statistical-reporting>)

**Response: Thank you for the question which is very complex since it contains many aspects. We provided response to each aspect of the question.**

1. **Please not that (Table 3), province was used as a unit of analysis. This means that analyses were done SEPARATELY for each province, and cross-tabulations were done between polygyny (which is a binary variable, coded “0” if the woman “*i*” from province “*j*” was in monogamous union, and “1”, otherwise) and “each type of violence (physical, emotional, and sexual)” or “intimate partner violence”. Results are reported for each province, statistical significance is reported using Chi-square test and p-values.**
2. **We provide an excerpt of what was done in the analysis for physical violence (Province of Kinshasa). The percentage should have been calculated in rows or columns. Since there were many calculations, we have calculated percentages in columns, to ease appending findings for all provinces. For instance, the excerpt below is for province “1” which represents Kinshasa in our coding. In the output, percentages are interpreted as “percentages of women who experienced physical violence among those in monogamous unions or polygynous families, respectively.**

**We have added notes in Table 3 to better guide readers on how to interpret figures in Table 3.**

**EXCERPT—1**

```
****************************************************************
***              PHYSICAL VIOLENCE                           ***


. display "This is for prov == 1"
This is for prov == 1

. tabout physical poly if fs & prov == 1 ///
>         using "$results/Sample.xls", ///
>         c(col) f(1) svy stats(chi2) npos(lab) percent replace
Survey results being calculated
1 ---+--- 2 ---+--- 3 ---+--- 4 ---+--- 5
.......

Table output written to: /Users/zacharietsaladimbuene_1/Desktop/DRC-DHSReports/IPV/Results/Sample.xls

In polygynous union
Physical violence       NO      YES     Total
                         %       %       %
NO (n=135)              86.2    74.3    85.5
YES (n=22)              13.8    25.7    14.5
Total (n=157)           100.0   100.0   100.0

Pearson: Uncorrected chi2(1) = 11.6609
Design-based F(1.00, 35.00) = 1.1108 Pr = 0.299
```

2. The data used to inform this study are over 10 years old, and may not be relevant to a contemporary context. Please be sure to discuss this as a limitation.

**Response: Thank you for this comment. We understand that data are aging a bit (10 years). However, it is important to note that (*i*) it is the most recent dataset available in the country at the time this study was undertaken; and (*ii*) more importantly, intimate partner violence is a heavy trend socially rooted in our communities. As such, it is not going to change in a morning.**

3. Please remove the final sentence of your conclusion, which is not directly relevant to the results presented here.

**Response: Thank you for the comment. This sentence was removed in the revised version.**
